# The role of participation and community mobilisation in preventing violence against women and girls: a programme review and critique

**DOI:** 10.1080/16549716.2020.1775061

**Published:** 2020-06-26

**Authors:** Nicole Minckas, Geordan Shannon, Jenevieve Mannell

**Affiliations:** Institute for Global Health, University College London, London, UK

**Keywords:** Community psychology, community action, participatory tools, gender, violence

## Abstract

**Background:**

Violence against women and girls (VAWG) is a public health problem and one of the most prevalent human rights violations in the world. Recently practitioners and researchers have taken an interest in community participation as a strategy for preventing VAWG. Despite the recent enthusiasm however, there has been little articulation of how participation in VAWG prevention programmes mobilises communities to challenge social norms and prevent VAWG.

**Objectives:**

In an attempt to help address this gap, this article seeks to answer two research questions: (1) How does participation theoretically mobilise communities to prevent VAWG, and (2) how do nominally *participatory* programmes make use of these theoretical concepts in their (explicit or implicit) theories of change?

**Methods:**

To answer the first question, we draw on two well-recognised theories of participation and community mobilisation – Rifkin and Pridmore’s continuum of participation and Freire’s steps towards achieving critical consciousness – to clarify theoretical assumptions about how participation can mobilise community to reduce VAWG. To answer our second research question, we present the results from a review of primary prevention programmes that seek to reduce VAWG through community participation. Our analysis examines the explicit and implicit theories of change for these prevention programmes against the assumptions outlined from the theoretical literature.

**Results:**

Our results help to better articulate realistic goals for community mobilisation and outline a theoretical basis for how participation as part of programming can effectively mobilise communities to reduce violence.

**Conclusion:**

We argue that, in order to be both effective and sustainable, the role of external agents in introducing programmes needs to be secondary to the ownership and empowerment of communities in designing and delivering their own strategies for VAWG prevention.

## Background

Violence against women and girls (VAWG) is a public health problem and one of the most prevalent human rights violations in the world [1]. It is estimated that approximately 30% of women will experience physical or sexual violence in their lifetime [2]. Given that VAWG is embedded in social contexts, prevention programmes require strategies that challenge the value systems, norms and social environments that normalise violence [3]. Community participation provides a means of challenging these social dynamics and has attracted widespread attention in recent years from both practitioners and researchers [4]. This article reviews the use of community participation as part of VAWG prevention programmes to develop better understandings of the theoretical assumptions these programmes make about how participation mobilises communities to reduce violence.

Over the past 15 years, a small number of discrete community-based interventions in low and middle income countries have been developed to challenge social norms that accept or condone acts of VAWG with some promising results [3,5,6]. For instance, the intervention *SASA*!, designed by the Ugandan non-profit organization *Raising Voices*, has reported changes in gender norms contributing to VAWG through training community-based activists to lead and facilitate community conversations about power relations, HIV/AIDS and violence against women [7]. *Stepping Stones* is another participatory community mobilisation intervention that has demonstrated changes in self-reported VAWG-related behaviours in short time frames with fewer men reporting the perpetuation of intimate partner violence, problem drinking and engagement in transactional sex after only two years [8]. Given the complex and deeply rooted causes and risk factors perpetuating violence, community participation in group activities such as these is said to help promote critical thinking about underlying inequalities between men and women, thereby encouraging community members to transform gender norms and prevent VAWG [9].

Despite these early successes, there has been little articulation in the VAWG field of how participation actually mobilises communities to change social norms or prevent violence. Although participation has been accepted as a key ingredient to address social norms *within* and *by* communities, it can also be easily misused and poorly applied [10–12]. As discussed by Cook (2012), there is a difference between *authentic* participation and mere involvement [13]. Whilst *authentic* participation refers to the ownership that comes with shared responsibility in the production of knowledge and improvement of practice, the term participation has been used – intentionally or not – to disguise top down implementation of externally designed programmes [14,15]. Scholars concerned about *authentic* participation oppose models in which communities are mere recipients rather than actual participants in the knowledge-development process [16]. This critical approach to participation places an emphasis on developing collaborative partnerships between different groups to foster empowerment and build people’s capacity to exercise greater agency over their well-being [17]. However, there is still a risk of shaping communities’ participation in order to fit the parameters of a pre-determined intervention, in ways that may reinforce hegemonic sources of knowledge [12]. Because participation can take on so many different meanings, there is an urgent need for a clear conceptual understanding of what participatory activities are trying to achieve for VAWG prevention interventions and how participation is understood to mobilise communities to reduce violence.

## Objectives

Therefore, in this article we review the use of community participation as part of VAWG prevention programmes for low- and middle-income countries in reference to the relevant theoretical literature on participation and community mobilisation. We do this in response to two research questions:

(1) How does participation theoretically mobilise communities to prevent VAWG?

(2) How do nominally *participatory* programmes make use of these theoretical concepts in their (explicit or implicit) theories of change?

## Methods

To understand how participation works to reduce violence theoretically (Q1), we draw on two of the best-recognised theories of participation and community mobilisation: Rifkin and Pridmore’s continuum of participation and Freire’s steps of consciousness-raising. This provides the theoretical basis for our review of nominally participatory VAWG prevention programmes, and their theories of change (Q2). Following our presentation of the theoretical literature, we summarise the methods we used to conduct our review of participatory VAWG prevention programmes and present the findings from our review. Following this, we discuss how our findings contribute to current understandings of participation and community mobilisation presented in the VAWG prevention literature and draw conclusions as to what this means for practitioners working in this field.

Our own definition of community participation draws on critical social theory, which places power relations at the centre of social analyses [18]. From this epistemological perspective, community mobilisation is a participatory and holistic process in which communities challenge the broader social and institutional structures that undermine collective efforts to prevent violence rather than only a means of changing social norms [19]. Participation mobilises community members to use their knowledge of social inequalities and vulnerability to build a collective response to health problems, thereby enabling greater self-reliance for the community while improving health outcomes [20]. Through this lens, it is the act of participating itself that helps individuals construct community identity, recognize structural reasons for violence, and take control over their lives [21].

### Theoretical framework: how participation mobilises communities

The vast majority of critical scholarship on community mobilisation draws on the work of Brazilian educationalist Paulo Freire [17]. Freire (1973) writes about the importance of developing an intellectual understanding of the social conditions that create disadvantage as a means of inspiring community groups to increase their sense of collective confidence and challenge or resist adverse social circumstances [22]. The endpoint of such process is what he calls ‘critical consciousness’. According to Freire, individuals start from a condition of intransitive thought in which they lack complete awareness of the social condition that undermines their wellbeing, and move towards intellectual autonomy, developing the ability to think holistically and critically about their condition (critical thinking). Once they have mastered the art of critical thinking, individuals are said to recognise the power of collective action to change social conditions and ability to improve their lives through a collective struggle (critical transitivity).

However, while promoting reflection and recognition of an adverse social reality is necessary, it is not a sufficient reason for communities to collectively take action towards changing that reality. Communities can be aware of the social structures that shape their lives, but their perceived lack of power can prevent them from taking control and challenging dominant structures [23]. For community mobilisation to take place, participants need to see themselves as the architects of their own lives. While Freire’s influential work on critical consciousness highlights the ways in which educational institutions have maintained oppression of the lower classes, it does not specify the specific practices that might empower or enable individuals to bring about change through their own actions. For this we turn to the literature on participation.

Scholars have long made the claim that participation is an empowering process, which can make individuals feel that they have control over the political, economic and psychological barriers to achieving change within their lives [24]. This understanding of participation as a lever of empowerment and therefore community mobilisation is consistent with Rifkin and Pridmore’s (2001) argument that community participation should move beyond people receiving information about the benefits of health programmes to people actively involved in decisions about policies and activities [25]. It is through this involvement in decision-making that individuals realise their potential to bring about change. Rifkin and Pridmore conceptualise participation as a continuum, which starts with the provision of information (information sharing) and finishes with the active process where intended beneficiaries influence programmes and grow personally (empowerment).

Rifkin and Pridmore’s continuum of participation is complementary and yet distinct from Freire’s process of critical consciousness. While both theoretical frameworks critique a ‘banking model of education’ (Freire, 1973) where information is given to recipients in a paternalistic fashion, they describe different processes for achieving social change. Freire’s framework is largely psychologically-oriented in describing how the process of thinking critically about the social conditions of one’s life creates the possibility for individuals to act collectively to change them. However, as discussed, this is insufficient on its own as even the most aware individuals may not have the confidence to bring about social change through collective action. Rifkin and Pridmore’s framework is largely action-oriented in describing how participation in decision-making helps individuals to see the possibilities for their actions to effect change. However, this is also insufficient on its own: participation in decision-making may be ineffective if the decisions taken are done so without an understanding of oppression. Both frameworks are therefore needed for a comprehensive understanding of how both critical consciousness (psychologically-oriented) and participation (action-oriented) contribute to the mobilisation of communities.

Therefore, we suggest that these two theoretical frameworks need to be brought together in order to understand how participation in programmes can mobilise communities to take collective action. It is not participation alone that allows this to happen, but rather participation combined with a process for achieving critical consciousness either alongside or as part of the participatory engagement.

**Figure 1.**
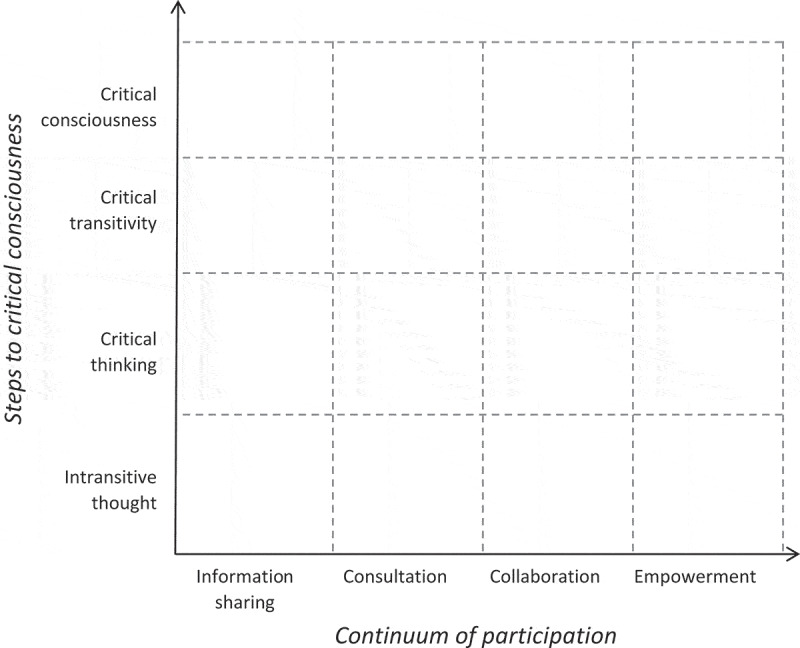
Theoretical framework for community mobilisation.

Figure 1 summarises this conceptualisation. The x-axis is constituted by the four levels of Rifkin’s continuum of participation model: information sharing, consultation, collaboration, and empowerment. The y-axis follows Freire’s steps of consciousness-raising from intransitive thought or ‘dependent thinking’ to ‘critical consciousness’. The space created between the two axes illustrates how increasingly meaningful participation contributes to higher levels of critical thinking and engagement in constructive action, with community mobilisation at the furthest end of the two axes.

This helps in thinking about how participatory VAWG prevention programmes should ideally be developing the individual capacity to think critically about the social conditions that contribute to violence, including institutionalised gender inequalities, systemic poverty, legal and policy frameworks that disadvantage survivors of violence – all of which have been identified as risk factors for VAWG [26]; while at the same time, involving individuals in making decisions about policy and activities so that they can see the potential for their actions to bring about broader social and institutional change. It is through this combined approach that community mobilisation is most likely to happen.

In response to the second research question posed in our introduction, we undertook a scoping review of the implicit and explicit theories of change developed as part of primary prevention programmes to reduce VAWG using community participation. We did this by first performing a rapid assessment of existing documents on participatory work for VAWG prevention by scanning Pubmed, Scopus and Google for published and unpublished literature. The initial aim of the search was to explore existing participatory train-the-trainer manuals for low-resource settings. Therefore, the search targeted programmes implemented in remote or low resource settings, without any restriction on geographical regions. The online search was done in May 2017 using (but not limited to) the following key words: rural population, rural health, violence, gender-based violence, violence against women, domestic violence, intimate partner violence, physical abuse, health promotion, primary prevention, community-based participatory research, participatory research, participatory action, VAW, GBV. This initial search yielded 47 documents focused on primary prevention of VAWG.

Simultaneously, we initiated a collaborative exchange via email with VAWG related-networks and international organisations to expand our search to a wider variety of sources. We included all organisations and networks that included the primary prevention of VAWG as part of their aims and objectives. We contacted 15 organisations and 37 scholars working with VAWG as a means of identifying relevant, non-published, tools. We publicized our search in social media, requesting for any tool developed through and/or for participatory activities focused on primary prevention of VAWG. We received 17 emails containing 23 toolkits in response to our request for evidence.

Our inclusion criteria for programme tools included interventions developed through a participatory community-based approach, implemented in low resource or remote settings, and with a thematic focus on the primary prevention of VAWG. We excluded the programme if the tools were not available online or upon request to the authors, there was not sufficient information to understand the theory of change for the programme, or if they were developed for implementation in high-income or urban settings. Of the limited number of primary prevention interventions found, only 28 had openly accessible toolkits. After screening, we ended up with 29 programmes that fulfilled our criteria. From those selected programmes, more than half (55%; n = 16) were carried out in African countries, 5 (17%) in an Asian country, 3 (10%) in Latin America and the remaining (17%, n = 5) across two or more regions. Table 1 summarizes the main characteristics of the selected toolkits.

**Table 1.** Characteristics of included programmes.

Programme	Country developed/implemented	Focus	Target population	Main activities
Abriendo Oportunidades (2004)	Guatemala	Girl empowerment and livelihood	Girls	Safe spaces, skill training
Access to Justed – Restless development (2013)	Sierra Leone	Gender-based violence awareness	Youth	Curriculum
Bantwana Initiative’s Pamoja Tuwalee (N/A)	Tanzania	Gender-based violence prevention	Community members	Dialogue sessions
Bell Bajao! (2008)	India	Domestic violence prevention	Youth	Media campaign and training
CARE’s Great Lakes Advocacy Initiative (GLAI)	Burundi, Rwanda and Uganda	Gender-based violence prevention	Community members	Advocacy
CHOICES (2009)	Nepal	Change gender norms	Youth	Workshop and discussion groups
Construyendo los avances de paz (N/A)	Bolivia	Violence against women prevention	Community members	Workshops
Deconstruyendo la Masculinidad	Honduras	Masculinities	Men	Workshop
Doorways/Abriendo puertas (2009)	Ghana and Malawi	School related Gender-based violence prevention	Youth	Educational curriculum
EA$E Programme (2014)	Cote D’Ivoire	Intimate Partner Violence Prevention	Married women	Group savings, skills training, group discussions
Engaging Men and Boys in Gender Equality and Health (2008)	Botswana, Brazil, Ghana, India, Kenya, South Africa, Swaziland, Tanzania, and Uganda	Masculinities, gender equality, HIV	Men all ages	Educational curriculum
IMAGE (2011)	South Africa	HIV and Gender equality	Rural women	Microfinance, Education sessions, community action plan development
Men’s Action to Stop Violence Against Women, MASVAW (2002)	India	Violence against women prevention, masculinities	Men	Workshops
Mobilising Men Initiative (2009)	India, Kenya, Uganda	Sexuall and Gender-based violence prevention	Men	Training and advocacy
One Man Can (N/A)	South Africa	Social justice, gender equality and engaged citizen activism	Men	Workshop
Promundo: Program HMD (2000)	Brazil	Gender equality	Youth	Workshop, campaigns
RESPOND/COMMPAC	Bolivia, Kenya	Post-abortion complications	Community members	Community action cycles with group session
Rwanda MoH National tool (2011)	Rwanda	Gender-based violence prevention	Community members	Workshop
SASA! (1999)	Uganda	Violence against women and HIV	Community members	Advocacy,multimedia, communication and training
Soul city (2005)	South Africa	Domestic violence prevention	General public	Social media
Stepping stone (1995)	Uganda	Sexual and reproductive health, HIV, gender and inter-generational relationships and rights.	Community members	Workshops
STOP (N/A)	Zambia	Women’s right and Gender-based violence awareness	Girls	Sports
Through our Eyes (2006)	Liberia, Rwanda, southern Sudan and Thailand	Gender-based violence, HIV/AIDS, harmful practices, and women and girls’ welfare	Members of conflict-affected communities	Participatory video
UNESCO: Promoting gender equality through community media among refugees in Ethiopia (2015)	Ethiopia	Gender based violence prevention	Community members	Media
VOICES project (N/A)	Nepal	Violence against women prevention	General Public	Community Radio
Voices4Change (2013)	Nigeria	Structural violence to gender equality	Adolescent girls and boys	Safe spaces, multimedia
We can campaign (2004)	Afghanistan, Bangladesh, India, Nepal, Pakistan and Sri Lanka	Domestic violence prevention	General public	Advocacy

**Figure 2.**
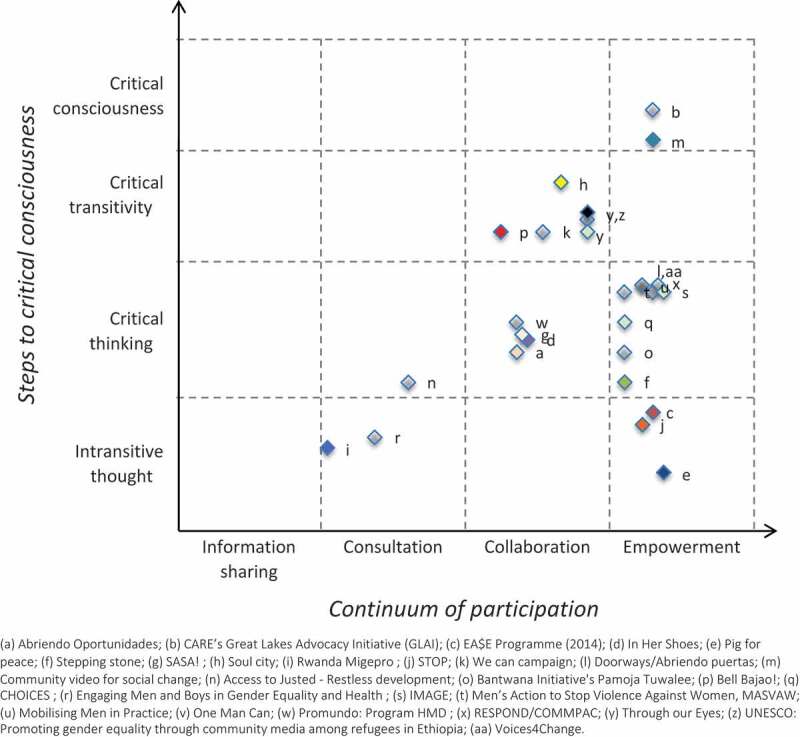
Allocation of tools within the theoretical framework (Supplementary Table 1).

Utilising the theoretical framework articulated in Figure 1, we undertook a framework analysis of the tools identified [27]. First, we extracted any text included in the tools that was associated with the theoretical approach taken to participation or community mobilisation. This broad approach to identifying relevant text allowed us to identify explicit ‘theories of change’ [28], as well as implicit understandings of how the programme could bring about social change and reduce VAWG that was mentioned in the text but not explicitly referred to as a theory of change. We then categorised these textual excerpts into overarching categories defined by the specific programme component described. These included: 1) problem identification; 2) developing the intervention; 3) information sharing; 4) decision-making; 5) implementation, 6) evaluation. After reaching team consensus on these categories, we scored each category for the individual programmes on a scale from 1 to 8 for both x and y axes (1 having the least amount of ‘participation’ or ‘consciousness-raising’, and 8 having the most). (Supplementary Table 1) A second researcher checked the results in order to ensure consistency of the scoring within categories. These scores were then used to plot the programmes into the matrix shown as shown in Figure 2.

## Results

Our review offers a critical assessment of the level of ‘participation’ (x-axis) and ‘consciousness-raising’ (y-axis) of different VAWG prevention programmes. We present the results of this review according to six main clusters described in terms of the intersecting axes on our matrix (see Figure 1), including: (1) intransitive thought – consultation, (2) intransitive thought – empowerment, (3) critical thinking – collaboration, (4) critical thinking – empowerment, (5) critical transitivity – collaboration, and (6) critical consciousness – empowerment. In each category, we present one or more participatory programmes identified to describe these clusters and their main characteristics.

### Intransitive thought – consultation

Programme documents that fit within this category follow top-down models of education, in which external agents identify an issue (here, VAWG) as a problem and share ‘dominant’ knowledge to shape participants’ understanding of it. We identified four toolkits that fit within this intersection between intransitive thought and consultation. These programmes were mainly framed within short training sessions or workshop addressed at increasing the knowledge about issues such as gender, masculinities, violence prevention strategies or legal concerns through didactic taught content. By way of illustration, Rwanda’s Ministry of Gender and Family Promotion’s (MIGEPROF) GBV prevention programme includes a knowledge evaluation section outlining ‘right/wrong’ or multiple-choice questions, showing the unidirectional nature of the programme, which in turn reduces its adaptability to local experiences, culture and norms [29]. An example of this type of multiple-choice verification is provided on page 17 of MIGEPROF’s manual:

“Circle all barriers preventing victims of gender-based violence to seek help:

a. Fear of consequences for themselves and their families’

b. The welcome received at Health Services, the police or the justice c. Modesty and shame to reveal

d. Economic Dependence

e. Lack of laws protecting victims and/or lack of knowledge of the existing laws protecting victims “

– Rwanda’s Ministry of Gender and Family promotion’s GBV training (page 17)

Although these toolkits mention a theoretical focus on encouraging reflection and promoting participation on issues related to VAWG, the prominent underlying characteristic of activities is an asymmetrical power/knowledge relationship between participants and facilitators. They include learning objectives such as explaining definitions, describing policies, categorising violence or building understanding on gender roles. They lack participation of communities in identifying the problem and developing a solution, which may increase dependency on external sources of information, perpetuating a state of intransitive thought, and reinforcing power imbalances.

### Intransitive thought – empowerment

Our analysis identified four tools that characterise the intersection of intransitive thought and empowerment. These include tools that provide a space for participants to engage in violence prevention only at an interpersonal or intrapersonal level or those that encourage economic empowerment. Although the level of consciousness remains low, participants are said to take a more active role within the programme, moving from mere recipients of information to essential actors, as illustrated in the following quote:

*“Members form and lead a community group that meets at least once a month to discuss challenges and successes [of the microfinance programme] and to guide new members for success” Pigs for Peace* [30]

Another example comes from the programme STOP – GENDER BASED VIOLENCE, one of several programmes that integrate sports with basic financial skills to promote awareness and encourage positive participation that can lead to a positive change [31]. This underlying change is expected to occur through skill-building activities that aim at tackling the root causes of VAWG and by promoting participation. Hence, it develops social and economic empowerment but without providing the space for reflection necessary for critical consciousness:

“From understanding health issues to learning basic financial skills, IN believes that sport can be an important tool in changing attitudes and helping young women improve their lives” STOP – GENDER BASE VIOLENCE IN/EduSport

Several of the programmes included in this category are microfinance programmes, such as the Economic and Social Empowerment programme (EA$E) [32]. The EA$E programme combines village savings and loans associations, business skill training and discussion group series. It is sustained by the idea that improving access to financial services will increase women’s economic empowerment:

“If women have access to financial services and increased and diversified sources of outcomes, and if men respect women and see them as valuable members of the household, then women will have a more equitable relationship and will experience a decrease in intimate partner violence” EA$E programme

Such programmes share the common characteristic of promoting action and skills learning over agency and reflection. The training modules revolve around leadership, development of saving, loans and social funds, record keeping, loan repayment, share-out of funds that will allow them to participate in micro loan groups. Therefore, participants’ sense of empowerment and participation increases, but the level of consciousness-raising remains low. Although they are the active ingredients, the process of problem recognition is provided by external sources and the drive to act remains at an individual or relational level. Participants are guided to uptake strategies to improve their current situation without acknowledging their responsibility within the broader social context or the root causes that affect the social reality of their life. They encourage a form of superficial empowerment to change their situation, while still placing responsibilities for the problem on others.

### Critical thinking – collaboration

Tools placed at the intersection of critical thinking and collaboration generally tend to share information through training programmes; however, they also provide a space for participants to analyse how that information is affecting their own life. Although this category encourages introspection and critical thinking, the degree of participation is reduced because of how the problem of violence is positioned within the tools. If the activities revolve around other people’s fictional stories of violence, they might encourage participants to reflect and analyse the complexity of the problem and its risk factors, while remaining an outsider. For example, the programme In Her Shoes, targeting both men and women, provides a space for participants to understand what it feels to be a woman, by discussing stories of women that had been victims of violence [33]. Thus, despite encouraging awareness and empathy, this toolkit still situates the problem in the shoes of others. That can be illustrated in the following quote:

“In her shoes is a learning exercise based on different women’s real-life experiences of violence. During this exercise, you will have the chance to spend time walking ‘in the shoes’ of these women and making the kinds of decisions with which they are faced” In her Shoes, page 16

The use of fictional stories is often used in VAWG training programmes as a means of avoiding potentially retraumatising experiences of telling one’s personal story of violence as part of a training or workshop activity. However, without any space for personal reflection on how experiences of violence relates to one’s own experience, the potential to encourage action towards social change is limited.

### Critical thinking – empowerment

Tools placed at the intersection of critical thinking and empowerment have a similar approach to critical thinking as the previous category, but also include a focus on authentic participation. These tools are often designed to alternate cycles of top-down education with spaces for reflection as with the programme *Stepping Stones*:

*“Stepping Stones workshops provide opportunities for participants to examine their values and attitudes towards gender and relationship, to build on their knowledge on aspects of sexuality and HIV/AIDS and to develop skills to help them communicate with others and ensure that other people know exactly what they want” Stepping Stones- creating future, page 54* [34]

The *Stepping Stone*s programme not only promotes critical thinking, but it also allows a space for reflection about the participant’s own life [34]. Therefore, it aims to translate the participant’s understanding of violence into their lived experience, allowing them to be critical of their action and aware of their position within society. This is accomplished in the toolkit by using activities such as personal stories discussions, medium-term goal setting and planning of their future. For example, the ‘Situating Self’ section of the Stepping-Stones Creating Future toolkit encourages participants to *‘ … reflect further on their life stories to reflect on the resources they draw on in building their lives and livelihood’ (Creating Future, page 28)*. In this way, the programme involves participants in designing strategies for reducing VAWG in their own lives and those around them.

An essential characteristic of the tools included in this category is their effort to develop a shared space to foster participation. For example, the toolkit for the programme *Abriendo Oportunidades* (Opening Opportunities), which creates safe spaces for girls and adolescents, describes an integrated curriculum that allows girls to take a look into their lives to identify the roots of the issues that limit their self-development and introduces the concept of ‘sisterhood’ as the main driver to ‘recognise shared challenges’ [35]. The power of groups and social connections are central to the tools included in this intersection, exceeding the sphere of the individual or relational, and focusing on the community and the social environment.

### Critical transitivity – collaboration

Tools included at the intersection of critical transitivity and collaboration tend to emphasise the value of community collaboration as part of the solution, thus removing the concept of violence as a private affair. Tool descriptions focus on mobilising communities through advocacy programs, or multimedia activities. However, they still require external agents to recognise the problem, to build alliances and to provide guidance to drive actions. An example of this is the *We Can Campaign* [36], which illustrates this in the first phase of its five phases theory of change:

“The aim of the initial phase is to increase awareness and promote reflection on violence against women, engaging the community to recognise violent practices as violence, and reflect on the root causes of discrimination and violence against women” (We can campaign, page 11)

The goal of these activities is to reach a wider audience, and to promote a shift in social attitudes and beliefs. It searches for such change by spreading the clear message that VAWG is unacceptable and equal relationships are violence-free. Another example is *Soul City*, a multi-media health promotion programme that utilises ‘edutainment’ (information linked to entertainment) through television series, radio drama, booklets, publicity and advocacy campaigns to ‘increase accurate knowledge’ and mobilise communities to take action on violence [37].

The commonality between the programmes in this category is the salience of collective action, alongside the remaining dependency on external support to encourage awareness and promote reflection. They are designed to have an impact at a socio-political level by building a collective that can envision a unified new future that can only be achieved by their actions.

### Critical consciousness – empowerment

Tools placed at the intersection of critical consciousness and empowerment place control of the programme into the hands of the community to promote collective ownership, foster resistance and challenge adverse social circumstances. The uniqueness characteristic of tools in this category is that the programmes maintain an equal distribution of knowledge between facilitators and participants. While the toolkits provide the channel, participants are responsible for identifying the problem of VAWG and bringing about their experiential knowledge to find a sustainable solution. Participants are the crucial actors and producers of reflection and subsequent change.

For example, the USAID programme *Community Video for Social Change* contains the fundamental principles of both critical consciousness and empowerment. In this toolkit, assistance from external partners is intended to support the technical aspects of recording and producing videos. The participatory nature of the activities are intended to directly increase the community’s decision-making power and advocacy skills in response to their local circumstances, as represented in the following quote:

“Community video is a communication approach that engages local people in creating videos that represent their lives and concerns. This approach is highly empowering because participants decide why and how to present different issues, what stories to tell, and how to represent themselves and their community. They also decide how the videos should be used and who should see them” (Community Video for Social Change, page 26)

Programmes in this category depend on the community to identify their needs, goals, and desired outcomes, emphasising self-representation to promote collective well-being. Another example is the Great Lakes Advocacy Initiative (GLAI) developed by CARE International. The toolkit for this programme provides grassroots activists with tools and information so that they can carry out ‘community-based advocacy’ [38]. The guide provides technical support on the how-to of advocacy campaigns in eleven steps, from choosing the issue related to VAWG through to developing advocacy objectives and actions, through to implementation and monitoring:

“An 11-step advocacy process is described in the following pages. The steps are:

Step 1: Choose the issue related to GBV

Step 2: Research and analyse the issue

Step 3: Identify key actors and institutions

Step 4: Analyse the policy environment

Step 5: Develop advocacy objectives

Step 6: Identify your target audience

Step 7: Identify your allies

Step 8: Choose your strategies/methods

Step 9: Develop key messages

Step 10: Create and implement your action plan

Step 11: Monitor”

(An advocacy Guide for Grass-root Activist in Burundi, page 15)

As mentioned previously, tools in this category are channels for communities to express their voices and to use the power of collective action to reflect on their social reality and to challenge it. The assumption underlying these programmes is that communities hold the knowledge of their social inequalities to build a collective response to their problems; they only need a space to enable reflection and the technical or financial support to turn it into action.

### Limitations

This study is an attempt to further knowledge and practice to higher levels of understanding on the relation between participation and community mobilisation, however it is not without its limitation. In order to take a pragmatic approach, we based our analysis on trainer manuals, which might have skewed the selection of the programmes, and therefore, our results, to more prescriptive programmes that required detailed and standardised procedures. In addition, our results might not reflect potential post hoc modifications that are inherent to participatory methodologies. Therefore, we could not illustrate the fluidity and flexible nature of participatory approaches.

## Discussion

Our review of how community participation is used as part of VAWG prevention programmes helps to identify the theoretical assumptions that underpin current programming in this field. While we approached this study from a conceptual standpoint, it has tangible practical implications for the development and evaluation of programmes. The theoretical framework illustrates the intersection between the process of developing critical consciousness (Freire) and community members’ level of participation (Rifkin and Pridmore), which provides a theoretical basis for designing participation VAWG prevention programmes. In reviewing the selected tools for VAWG prevention, we have identified different mechanisms by which programmes aim to encourage participation and social changes that reduce violence. This lends itself to identifying specific activities that might be beneficial additions to programmes.

Broadly speaking, programmes that consist purely of a unidirectional educational component do not encourage critical consciousness, nor do they empower participants to make changes through collective action; whereas programmes that collaborate with communities to design activities and programme elements increase communities’ sense of ownership over the intervention itself. However, there are many activities that fall between these two extremes. The process of building critical consciousness or empowering individuals to take collective action may be more incremental. The programmes we reviewed included activities with extraordinary potential, including: creating spaces for critical reflection through question sessions, open discussions and debates about social reality; using personal stories to reflect on lived experiences; and creating safe mentor-led spaces for girls. While our theoretical framework outlines how community mobilisation occurs when community members have already recognized themselves as active players in their social reality and are ready to bring about change, our results highlight the range of activities for achieving this objective. There is not a one-size fits all approach to achieved critical consciousness and empowerment – it is through a patchwork of activities and ideas (albeit with a shared theory of change) that this may be achieved. This resonates with Hatcher et al.’s [39] evaluation of the IMAGE trial in South Africa and their observation that activities supporting Freirian *conscientisation* as an end-point are often not linear and have feedback loops that bring participants back to reflection after participating in action.

Another learning from our results is the distinct difference between knowledge and awareness. Knowledge can be understood as factual information acquired from authoritative external sources [40] and can reinforce an asymmetrical power dynamic promoting dependent thinking [41]. However the concept of awareness relates knowledge to people’s own reality, producing a transitory understanding and a sense of rejection of the status quo [42]. Therefore, programmes focused around increasing knowledge alone (such as pure educational campaigns) constrain programme objectives for community mobilisation. In particular, in many settings violence is normalised and communities’ reactions to prescriptive messages can backlash into defensiveness, confusion, and overall rejection [43]. Compared to this, it can be expected that activities that are less prescriptive in nature (awareness raising activities) provide a larger space for reflection on the social roots that influence communities’ marginalised position. This link between knowledge and awareness represented in the framework can help to point out the level of participation being promoted by the programmes, and how (or to what extent) it can potentially push people towards a higher level of consciousness.

In the same way as knowledge is not equivalent to awareness, action should not be seen as equivalent to mobilisation. Many programmes designed on an action-led notion of change do not necessarily provide sufficient space for developing collective agency, and do not address contextual and structural drivers. Programmes such as cash transfers, in which women use the tools provided to increase their families’ wellbeing, or sports activities that promote spaces for girls to share common interests or concerns, follow an action-led notion of change. Their underlying assumption is that mobilisation occurs by simply enabling an environment or giving material/financial means for groups to come together and feel empowered, with little attention to other contextual system drivers [17,44]. However, following Freire’s ideas, community mobilisation occurs when community members have developed a collective sense of agency that can sustainably challenge the status quo [22]. Therefore, we highlight the notion that communities mobilise when they are inherently empowered and aware of the social conditions that constrain their full potential, limiting the power of external agents to trigger this action.

Perhaps the most important implication of our framework is that it helps to situate VAWG prevention programmes within a spectrum of participation and critical consciousness, so that current and future programmes can better articulate realistic goals and ensure a clear progress towards more effective community engagement in VAWG prevention. It emphasises the importance of participatory methodologies to encourage communities to move towards collective sense of agency and action, and challenges more mechanistic, top-down, and unidirectional strategies. By considering and critiquing interventions using critical theory, we are able to rethink interventions’ effectiveness and promote a pragmatic approach for identifying real outcomes that can be obtained according to the type of activities proposed by the programme.

Further, our approach has attempted to unmask the use of participation as a mechanism to achieve community mobilisation based on the risk of imposing an implicit agenda by pre-defined messages or activities by external agents. The corruption of the concept of participation through an outsider’s agenda can hinder the empowering process of change within people, and the growth of authentic participation [24]. Our insights are consistent with others who have highlighted the misappropriation of nominally participatory methods in global health and international development. Nichter (1984) warns of the absence of critical, sociocultural perspectives in community participation and bottom-up planning, and called for the de-professionalization of social science research [45]. Cornwall and Pratt (2011) outline a sort of legacy language used by development actors referring to ‘civil society participation’ or ‘social accountability,’ without meaningful grassroots participation [46]. Conchelos and Kassam (1981) also highlight the misuses of participatory research through a lip-service to ‘formal participation’ and summarises how accidental misuse of participatory approaches may lead to a ‘sophisticated oppression’ of those intended to benefit from the process [47].

Overall, our results challenge whether, in fact, external agents can promote community mobilisation, or if communities can only achieve mobilisation when they are sufficiently (*self*)empowered and aware of the social conditions that limit their full potential. In other words, when communities become aware of their marginalised role within society and are able to construct a new narrative to achieve emancipatory social change.

## Conclusion

In conclusion, developing strategies to prevent VAWG is still a challenge. Participatory approaches and community mobilisation appear to be a way forward for VAWG prevention, but the concepts remain unclear and evidence is scarce. This paper aimed to open the black box by presenting a framework to analyse how the intersection between critical consciousness (psychologically-oriented) and participation (action-oriented) influence community mobilisation. Through our analysis, we conclude that communities must be intrinsically aware and empowered to achieve critical consciousness and to mobilise for sustainable change on VAWG prevention. The recurrent – and ever more frequent- goal of researchers and decision-makers to encourage communities to mobilise against gender violence is in many ways paradoxical. Researchers can support knowledge on risk factors and promote awareness on gender norms, but, ultimately, it is in the hands of the communities to identify VAWG as their struggles and to mobilise against the systemic social norms in which they live to bring about change.

## Supplementary Material

Supplemental Material

## Paper context

Violence against women and girls (VAWG) is a public health problem and one of the most prevalent human rights violations in the world. Participatory approaches and community mobilisation appear to be a way forward for violence prevention, but their concepts remain unclear and evidence is scarce. This paper aims to open the black box by presenting a framework to analyse how the intersection between cognitive-emotional development and participation influence community mobilisation for VAWG prevention.

## References

[1] Krug EG, Mercy JA, Dahlberg LL, et al. The world report on violence and health. Lancet. 2002;360:1083–12. 12384003 10.1016/S0140-6736(02)11133-0

[2] World Health Organization. WHO multi-country study on women’s health and domestic violence against women: initial results on prevalence, health outcomes and women’s responses. Geneva, Switzerland: WHO Press; 2005.

[3] Jewkes R. Intimate partner violence: causes and prevention. Lancet [Internet]. 2002;359:1423–1429. Available from: https://www.ncbi.nlm.nih.gov/pubmed/11978358 11978358 10.1016/S0140-6736(02)08357-5

[4] Heise LL What works to prevent partner violence? An Evidence Overview Working paper (version 2.0). 2011.

[5] Ellsberg M, Arango DJ, Morton M, et al. Prevention of violence against women and girls: what does the evidence say? Lancet [Internet]. 2015;385:1555–1566. Available from: https://www.ncbi.nlm.nih.gov/pubmed/25467575 25467575 10.1016/S0140-6736(14)61703-7

[6] Kyegombe N, Starmann E, Devries KM, et al. “SASA! Is the medicine that treats violence”. Qualitative findings on how a community mobilisation intervention to prevent violence against women created change in Kampala, Uganda. Glob Health Action [Internet]. 2014;7:25082. Available from: https://www.ncbi.nlm.nih.gov/pubmed/25226421 25226421 10.3402/gha.v7.25082 PMC4165071

[7] Abramsky T, Devries K, Kiss L, et al. Findings from the SASA! Study: A cluster randomized controlled trial to assess the impact of a community mobilization intervention to prevent violence against women and reduce HIV risk in Kampala, Uganda. BMC Med [Internet]. 2014 cited 2020 57;12:122. Available from: http://bmcmedicine.biomedcentral.com/articles/10.1186/s12916-014-0122-5 25248996 10.1186/s12916-014-0122-5 PMC4243194

[8] Jewkes R, Nduna M, Levin J, et al. Impact of stepping stones on incidence of HIV and HSV-2 and sexual behaviour in rural South Africa: cluster randomised controlled trial. BMJ. 2008;337:391–395. 10.1136/bmj.a506 PMC2505093 18687720

[9] Michau L, Horn J, Bank A, et al. Prevention of violence against women and girls: lessons from practice. Lancet. 2015;385:1672–1684. 25467577 10.1016/S0140-6736(14)61797-9

[10] Leal PA. Participation: the ascendancy of a buzzword in the neo-liberal era. Dev. Pract. 2007;17:539–548.

[11] Rifkin SB, Kangere M What is participation. Community-based Rehabil. As a Particip. Strateg. Africa. 2002;37–49.

[12] Vijayakumar G. Collective demands and secret codes: the multiple uses of “community” in “community mobilization.”. World Dev [Internet]. 2018;104:173–182. Available from: http://www.sciencedirect.com/science/article/pii/S0305750X17303716

[13] Cook T. Where participatory approaches meet pragmatism in funded (health) research: the challenge of finding meaningful spaces. Forum Qual. Sozialforsch./Forum Qual. Soc. Res. Particip. Qual. Res. [Internet]. 2012;13. Available from: http://www.qualitative-research.net/index.php/fqs/article/view/1783/3305

[14] McTaggart R. Guiding principles for participatory action research. In: Participatory action research: International contexts and consequences. 1st ed. New York, USA: Suny Press; 1997. p 25–43.

[15] Cornwall A, Brock K. Beyond buzzwords “poverty reduction”,“participation” and “empowerment” in development policy. Geneva, Switzerland: UNRISD; 2005.

[16] Ortiz L. Toward authentic participatory research in health: a critical review. Pimatisiwin. 2003;1:1–26.

[17] Campbell C. Community mobilisation in the 21st century: updating our theory of social change? J Health Psychol [Internet]. 2014 cited 2020 57;19:46–59. Available from: http://journals.sagepub.com/doi/10.1177/1359105313500262 24000384 10.1177/1359105313500262

[18] Held D. Introduction to critical theory: horkheimer to Habermas. California, USA: University of California Press; 1980.

[19] Mannell J, Dadswell A. Preventing intimate partner violence: towards a framework for supporting effective community mobilisation. J. Community Appl. Soc. Psychol. 2017;27:196–211.

[20] Campbell C, Cornish F, Gibbs A, et al. Heeding the push from below: how do social movements persuade the rich to listen to the poor? J. Health Psychol. 2010;15:962–971. 20631042 10.1177/1359105310372815

[21] Campbell C, Cornish F. Towards a “fourth generation” of approaches to HIV/AIDS management: creating contexts for effective community mobilisation. AIDS Care. 2010;22:1569–1579. 21161761 10.1080/09540121.2010.525812

[22] Freire P. Education for critical consciousness: bloomsbury academic [Internet]. Paulo Freire Read. 1974 cited 2020 57. Available from: https://www.bloomsbury.com/uk/education-for-critical-consciousness-9781780937816/

[23] Campbell C, Jovchelovitch S. Health, community and development: towards a social psychology of participation. J. Community Appl. Soc. Psychol. 2000;10:255–270.

[24] Rahnema M. Participatory action research: the “last temptation of saint” development. Altern. Glob. Local, Polit. [Internet]. 1990 cited 2020 57;15:199–226. Available from: http://journals.sagepub.com/doi/10.1177/030437549001500204

[25] Rifkin S, Pridmore P. Partners in planning: information, participation and empowerment. London, UK: Macmillan; 2001.

[26] Gibbs A, Dunkle K, Ramsoomar L, et al. New learnings on drivers of men’s physical and/or sexual violence against their female partners, and women’s experiences of this, and the implications for prevention interventions New learnings on drivers of men’s physical and/or sexual violence against their female partners, and women’s experiences of this, and the implications for prevention interventions. Glob Health Action. 2020 cited 2020 57. DOI:10.1080/16549716.2020.1739845. PMC7144308 32202227

[27] Gale NK, Heath G, Cameron E, et al. Using the framework method for the analysis of qualitative data in multi-disciplinary health research. BMC Med Res Methodol [Internet]. 2013 cited 2020 57;13:117. Available from: https://bmcmedresmethodol.biomedcentral.com/articles/10.1186/1471-2288-13-117 24047204 10.1186/1471-2288-13-117 PMC3848812

[28] De Silva MJ, Breuer E, Lee L, et al. Theory of change: A theory-driven approach to enhance the medical research council’s framework for complex interventions. Trials [Internet]. 2014 cited 2020 57;15:267. Available from: https://trialsjournal.biomedcentral.com/articles/10.1186/1745-6215-15-267 24996765 10.1186/1745-6215-15-267 PMC4227087

[29] MiGeProf. Gender based violence training [Internet]. Rwanda: Ministry of Gender and Family Promotion; 2011. Available from: https://migeprof.gov.rw/fileadmin/_migrated/content_uploads/GBV_Training_MODULE_English_Version.pdf.

[30] PFP Congo. Pigs for peace [Internet]. DRC: Promotion de la Famille Paysanne; 2011. Available from: https://www.pfpcongo.com/en-home

[31] International Inspiration. STOP GBV [Internet]. UK: Department for International Development; 2013. Available from: http://www.internationalinspiration.org/women-and-girls

[32] International Rescue Committee. Programme EA$E [Internet]. UK: International Rescue Commitee; 2014. Available from: https://gbvresponders.org/empowerment/eae-tools-resources/

[33] Raising voices, Voices TGBVPN and R. In her shoes: a toolkit for reflecting on violence against women in Sub-Saharan Africa [Internet]. Perspectives R, editor. Earn. From Pract. Ser. Uganda; 2011. Available from: http://raisingvoices.org/resources/.

[34] Salamander Trust. Stepping stones project [Internet]. Uganda: Salamander Trust; 2011. Available from: https://steppingstonesfeedback.org/resources/gender-based-violence/

[35] Population Council. Abriendo Oportunidades [Internet]. New York, USA: Population Council; 2019. Available from: https://www.popcouncil.org/about/leadership#Country_Directors

[36] Oxfam. We can campaign. UK: Oxfam; 2018.

[37] Usdin S, Scheepers E, Goldstein S, et al. Achieving social change on gender-based violence: a report on the impact evaluation of Soul City’s fourth series. Soc Sci Med [Internet]. 2005;61:2434–2445. Available from: https://www.ncbi.nlm.nih.gov/pubmed/16006028 16006028 10.1016/j.socscimed.2005.04.035

[38] CARE. Great Lakes Advocacy Initiative (GLAI) [Internet]. Burundi: CARE; 2011. Available from: https://www.care.org/sites/default/files/documents/GBV-2012-Advocacy-guide-for-grassroots-activists-for-GBV-Burundi.pdf

[39] Hatcher A, De Wet J, Bonell CP, et al. Promoting critical consciousness and social mobilization in HIV/AIDS programmes: lessons and curricular tools from a South African intervention [Internet]. Health Educ Res. 2011 cited 2020 57; 542–555. Available from: https://academic.oup.com/her/article/26/3/542/735899 20965911 10.1093/her/cyq057

[40] Trevethan R. Deconstructing and assessing knowledge and awareness in public health research. Front Public Health. 2017;5:194. 10.3389/fpubh.2017.00194 PMC5545880 28824900

[41] Chambers R. Institute of development studies (Brighton E. Paradigm shifts and the practice of participatory research and development. Brighton, UK: Institute of Development Studies; 1994.

[42] Bandura A. Social cognitive theory of mass communication. Media Eff. Routledge. 2009;3:110–140.

[43] Michau L. Approaching old problems in new ways: community mobilisation as a primary prevention strategy to combat violence against women. Gend. Dev. [Internet]. 2007;15:95–109. .

[44] Moeller K. Proving “The Girl Effect”: corporate knowledge production and educational intervention. Int. J. Educ. Dev. 2013;33:612–621.

[45] Nichter M. Project community diagnosis: participatory research as a first step toward community involvement in primary health care. Soc Sci Med. 1984;19:237–252. 6484613 10.1016/0277-9536(84)90215-6

[46] Cornwall A, Pratt G. The use and abuse of participatory rural appraisal: reflections from practice. Agric. Human Values. 2011;28:263–272.

[47] Conchelos G, Kassam Y. A brief review of critical opinions and responses on issues facing participatory research. Convergence. 1981;14:52.

