# Supplementary material for: The role of participation and community mobilisation in preventing violence against women and girls: a programme review and critique
Source: Glob Health Action. 2020 Jun 26;13(1):1775061. doi: 10.1080/16549716.2020.1775061 (PMC7480621; doi:10.1080/16549716.2020.1775061)
Supplement: Supplemental Material [file ZGHA_A_1775061_SM7545.docx]

**Supplementary table 1. Score of identified tools**

| **Tool/programme** | **In-graph reference** | **Continuum of participation (x-axis)** | **Critical consciousness (y-axis)** |
| --- | --- | --- | --- |
| Abriendo Oportunidades | a | 4.5 | 3.0 |
| CARE’s Great Lakes Advocacy Initiative (GLAI) | b | 7.0 | 7.0 |
| EA$E Programme | c | 7.0 | 2.0 |
| In Her Shoes | d | 4.5 | 3.0 |
| Pig for peace | e | 7.0 | 1.0 |
| Stepping stone | f | 6.5 | 2.5 |
| SASA! | g | 4.5 | 3.5 |
| Soul city | h | 5.5 | 6.0 |
| Rwanda Migepro | i | 1.0 | 1.5 |
| STOP | j | 7.0 | 2.0 |
| We can campaign | k | 5.0 | 5.0 |
| Community video for social change | m | 7.0 | 6.5 |
| Access to Justed - Restless development | n | 2.5 | 2.5 |
| Bantwana Initiative's Pamoja Tuwalee | o | 6.5 | 3.0 |
| Bell Bajao! | p | 4.0 | 5.0 |
| CHOICES | q | 6.5 | 3.5 |
| Engaging Men and Boys in Gender Equality and Health | r | 2.0 | 1.5 |
| IMAGE | s | 7.0 | 4.0 |
| Men’s Action to Stop Violence Against Women, MASVAW | t | 6.5 | 4.0 |
| Mobilising Men in Practice | u | 7.0 | 4.0 |
| One Man Can | v | 6.0 | 5.5 |
| Promundo: Program HMD | w | 4.5 | 3.5 |
| RESPOND/COMMPAC | x | 7.0 | 4.0 |
| Through our Eyes | y | 6.0 | 5.0 |
| UNESCO: Promoting gender equality through community media among refugees in Ethiopia | z | 6.0 | 5.0 |
| VOICES project | aa | 4.5 | 5.0 |
| Voices4Change | ab | 7.0 | 4.0 |
| Deconstruyendo la Masculinidad | ac | 2.0 | 2.0 |
| Doorways/Abriendo puertas | ad | 7.0 | 4.0 |
